# Supplementary material for: Murine Mast Cells That Are Deficient in IFNAR-Signaling Respond to Viral Infection by Producing a Large Amount of Inflammatory Cytokines, a Low Level of Reactive Oxygen Species, and a High Rate of Cell Death
Source: Int J Mol Sci. 2023 Sep 15;24(18):14141. doi: 10.3390/ijms241814141 (PMC10531704; doi:10.3390/ijms241814141)
Supplement: Supplementary file 1 [file ijms-24-14141-s001.zip › ijms-2566893-supplementary.pdf]

Supplementary Information

Material and Methods

Cytospin preparation of BMMCs and Wright-Giemsa staining to analyze the cells

To conduct an experiment to analyze cell morphology, BMMCs were left in media or treated with rVSVΔm51 at MOI 10 and 50 for 20 hours. After harvesting, washing, and resuspending the cells in PBS, they were centrifuged onto a circular area on a slide cytospin to create a monolayer of cells. Wright-Giemsa stain was used to color the cells, which were then examined under a light microscope for analysis of their mor-phology and degranulation.

Influence of N-acetyl-L-cysteine (NAC) on Cellular Apoptosis

1×105 BMMCs were cultured in a complete RPMI medium for 2 days, with or without the addition of 10 μM NAC. The BMMCs were incubated for 4 hours at 37°C, followed by exposure to rVSVm51 at an MOI of 10. After the 4-hour incubation, prior to analysis using the BD FACS-CANTO II flow cytometer, cells were stained with 7AAD for 20 minutes to assess cell viability.

Results

BMMCs Infected with rVSVΔm51 Exhibit Observable Degranulation

BMMCs contain large numbers of preformed mediators that are released in response to stimuli. To assess degranulation in BMMCs with and without the IFNAR signaling, the cells were exposed to rVSVΔm51 at MOI 10 and 50 for 20 h and then stained with Wright-Giemsa. Light microscopy analysis revealed significant degranulation in IFNAR−/− BMMCs after exposure to rVSVΔm51. The low number of MCs with intact morphology within IFNAR−/− BMMCs suggests a high rate of cell death, consistent with the results obtained from flow cytometry analysis and the resazurin assay.

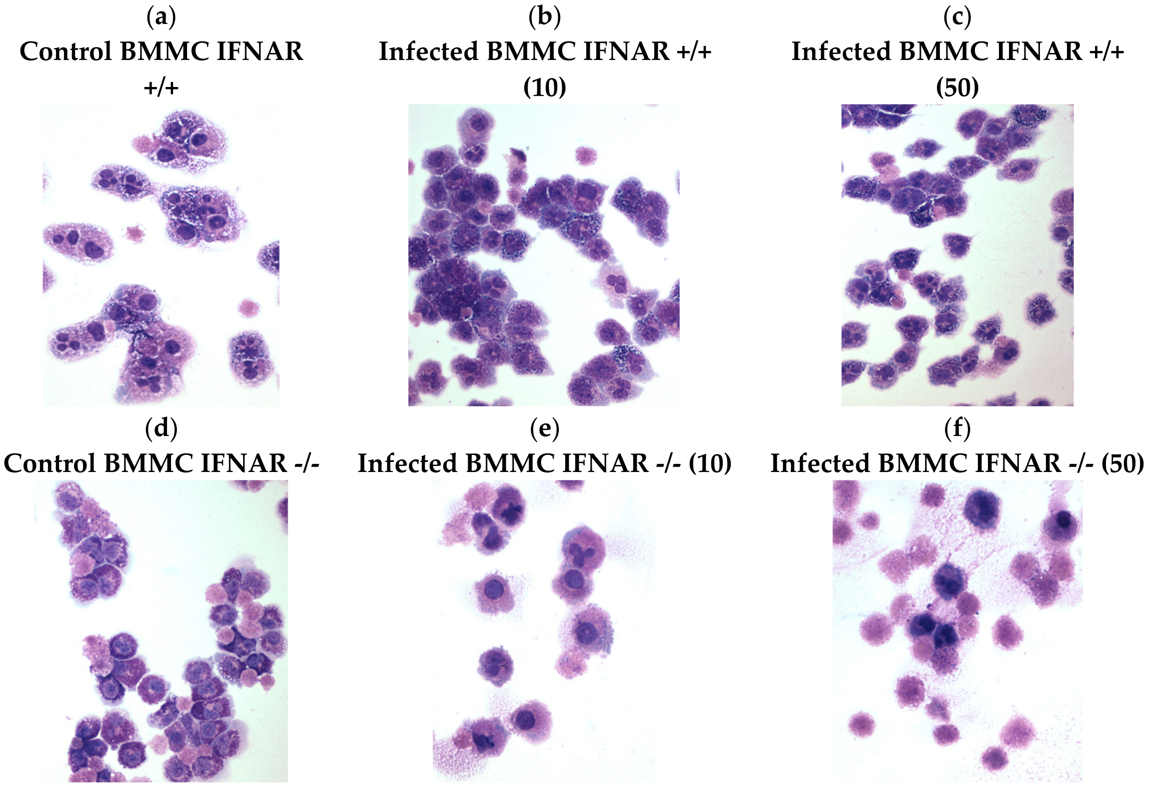

Figure S1. BMMCs infected with rVSVΔm51 exhibit observable degranulation. BMMCs were left untreated or infected with rVSVΔm51 and then stained with Wright-Giemsa to visualize their morphology by light microscopy. (a) IFNAR +/+ BMMCs, (b) infected IFNAR +/+ BMMCs at MOI 10 and (c) MOI 50, (d) IFNAR -/- BMMCs, (e) an infected IFNAR -/- BMMCs at MOI 10, and (f) MOI 50 after 20 hours. Each image is a microscopic view of a Giemsa-Wright stained BMMC, magnified at x40.

NAC Modulates Cell Viability of BMMCs After Infection with rVSVΔm51

A total of 1×10^5 BMMCs were cultured in a complete RPMI medium for two days, with or without the addition of 10 µM NAC prior to exposure to rVSVm51 at an MOI of 10. The BMMCs IFANR+/+ and IFNAR−/− treated with NAC had more live cells than the control group that was only exposed to media before treatment with rVSVm51. This suggests that NAC treatment can increase cell survival in infected BMMCs.

(**a**)

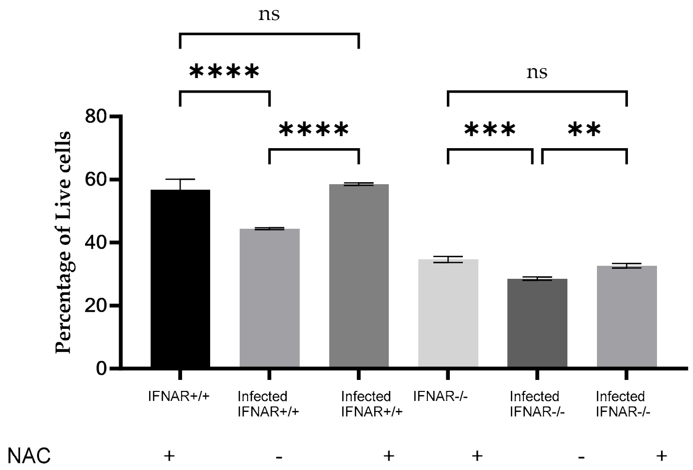

(**b**)

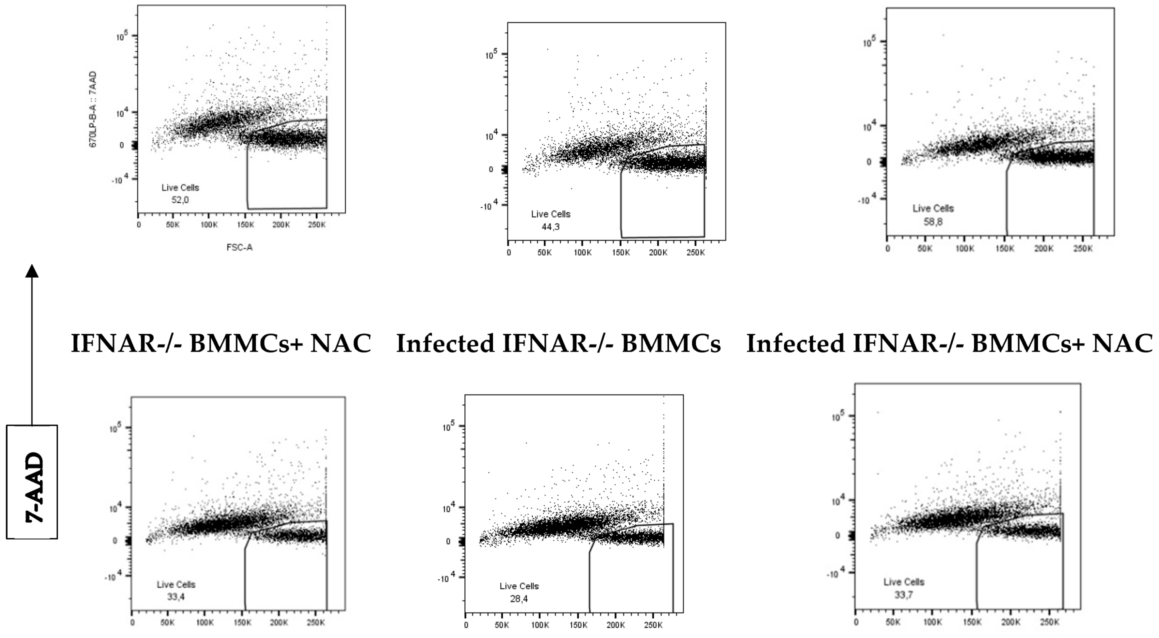

Figure S2. NAC modulates cell viability of BMMCs after infection with rVSVΔm51. BMMCs were cultured in a complete RPMI medium for 2 days in the presence or absence of NAC (10 µM). The BMMCs were exposed to rVSVm51 at a multiplicity of infection (MOI) of 10 for 4 hours. After harvesting, the cells were stained with 7-AAD and analyzed using flow cytometry to detect dead cells. (a) Graphs show the percentage of live cells after exposure to rVSVΔm51. Statistical significance was determined using a one-way ANOVA with Tukey's multiple comparison test. (b) Representative flow cytometry dot plot graphs showing the percentage of live cells that are 7-AAD negative.
